# Supplementary figures and images for: Interpretable machine learning for predicting isolated basal septal hypertrophy
Source: PLoS One. 2025 Jun 30;20(6):e0325992. doi: 10.1371/journal.pone.0325992 (PMC12208500; doi:10.1371/journal.pone.0325992)

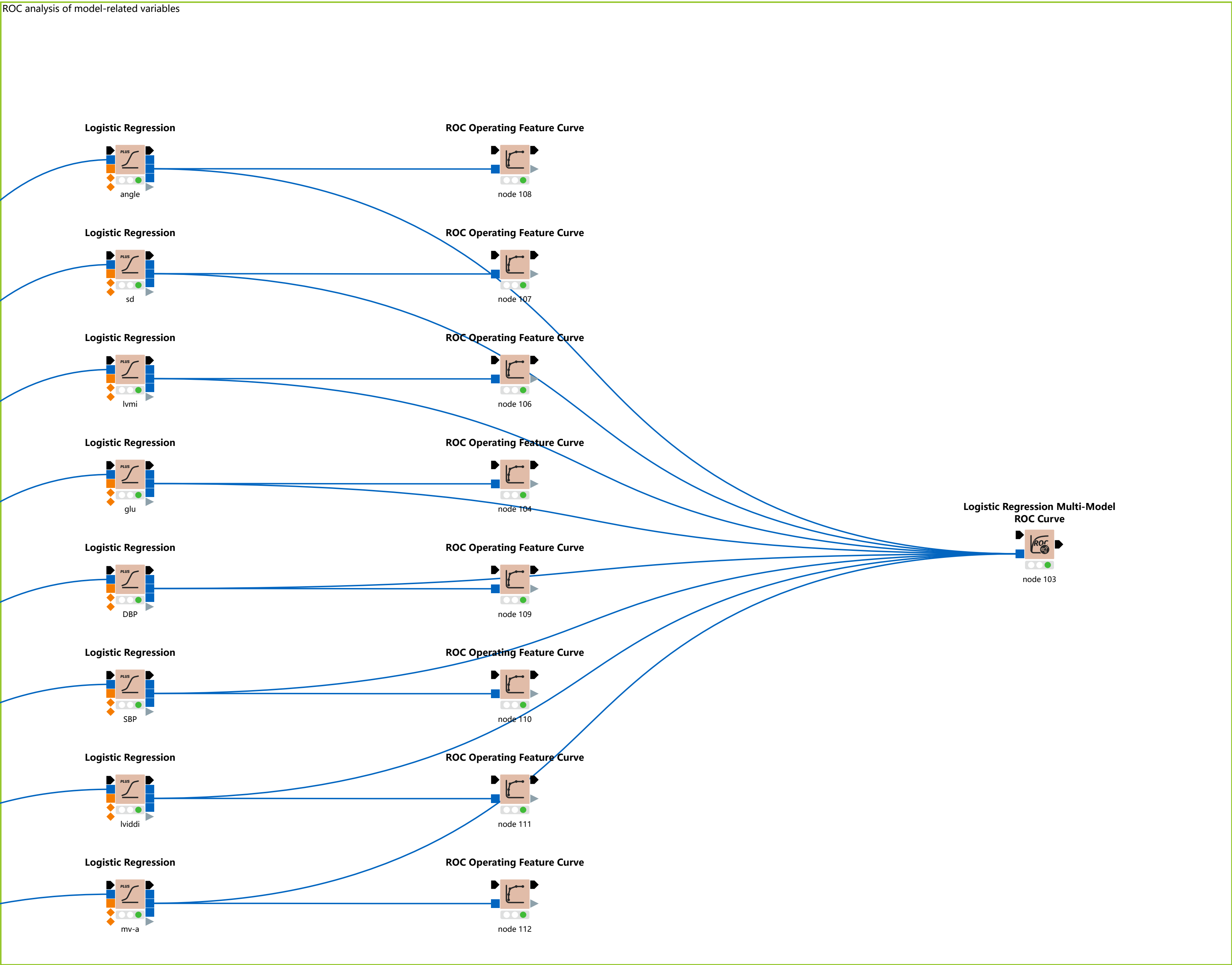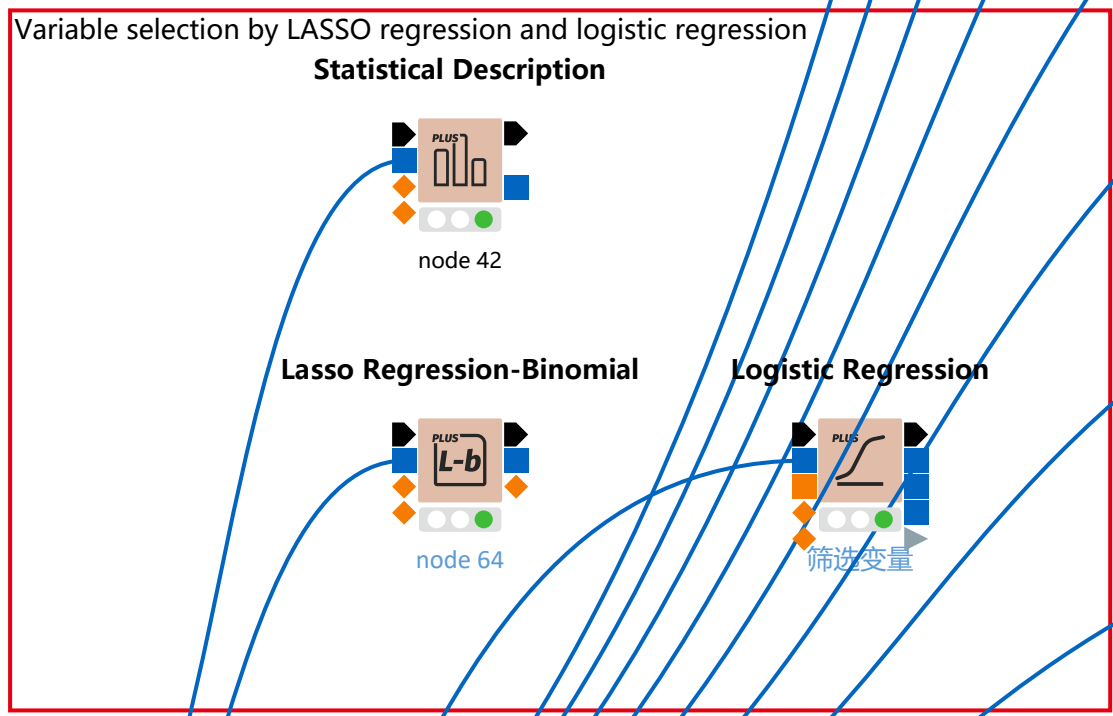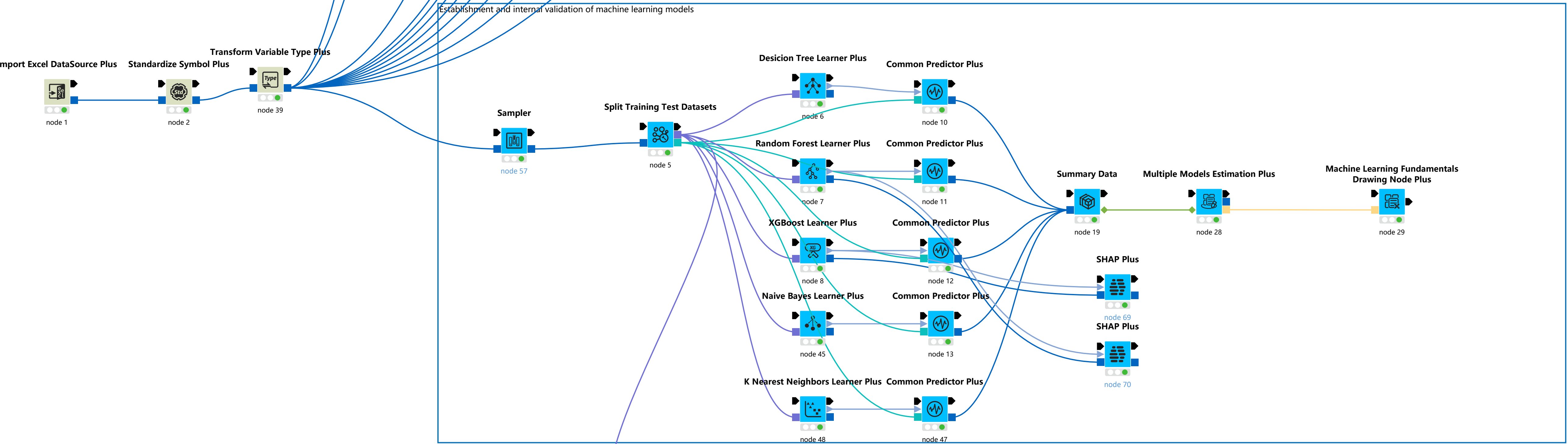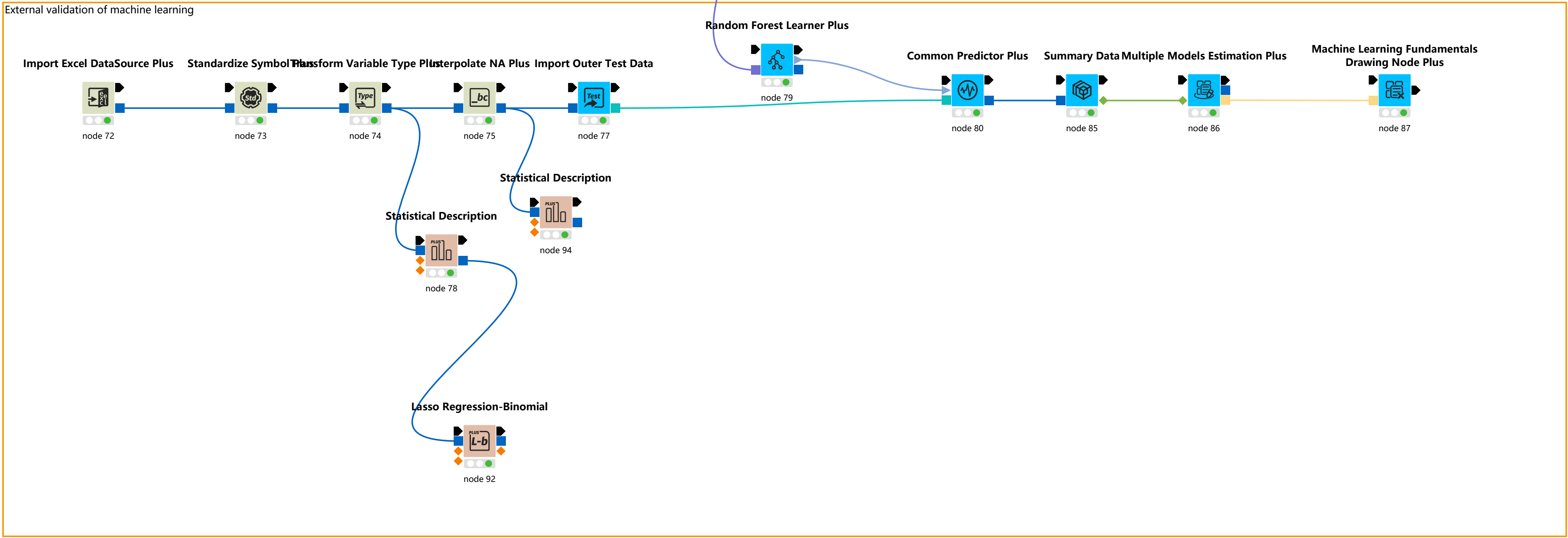

Supplement: S4 File — (PDF) [file pone.0325992.s004.pdf]
