# Supplementary material for: Interpretable machine learning for predicting isolated basal septal hypertrophy
Source: PLoS One. 2025 Jun 30;20(6):e0325992. doi: 10.1371/journal.pone.0325992 (PMC12208500; doi:10.1371/journal.pone.0325992)
Supplement: Attachment — Submitted filename: Response to Reviewers.docx [file pone.0325992.s007.docx]

Response to Reviewers

Manuscript ID number:

PONE-D-24-57853

Title of paper:

Interpretable Machine Learning for Predicting Isolated Basal Septal Hypertrophy

Dear Editor and Reviewers,

Thank you for your meticulous review and valuable suggestions on our manuscript. Below is our point-by-point response to the comments:

**1. Regarding Journal Requirements: Reference List Check**

We have conducted a rigorous review of the reference list and uniformly reformatted all citations into Vancouver style as per journal requirements. All cited references have been confirmed to be indexed in PubMed, with no inclusion of retracted articles. The completeness and accuracy of the references have been systematically validated according to the journal’s guidelines.

**2. Response to Reviewer 1’s Comments**

Reviewer 1 stated: “I would like to thank the authors for their adequate and to-the-point responses. I recommend using Figures 1, 2, and 4.”
**Response:**
We appreciate your acknowledgment of our responses. We fully accept your recommendation on the use of figures and have incorporated Figures 1, 2, and 4 in the revised manuscript. These figures now clearly present key results. We are happy to optimize any details further if needed.

**3. Response to Reviewer 2’s Comments**

1. **Correction of Spelling Error**
   Reviewer 2 noted: “Page 14, line 274: ‘tudied’ should be corrected to ‘studied’.”
   **Response:**
   Thank you for identifying this oversight. We have corrected the spelling error in the revised manuscript (Page 14, Line 274) and conducted a comprehensive language proofread to ensure accurate and clear expression throughout the text.
2. **Addition of Future Research Recommendations Section**
   **Response:**
   We have adopted your suggestion and added a new subsection titled “Future Research Recommendations” at the end of the discussion section (new content on Page 16, Lines 307-315). This section outlines two directions, including exploring the application of interpretable algorithms in BSH with left ventricular outflow tract obstruction, and adding a temporal dimension to address the limitations of the study and guide future research directions.

Once again, we thank the Editor and Reviewers for your professional insights. We believe the revised manuscript now meets the journal’s publication criteria. Please let us know if any further adjustments are needed, and we will gladly cooperate.

Best regards,
Yueheng Wang
26500167@hebmu.edu.cn
Month 16, 2025
